# Analyzing the lncRNA, miRNA, and mRNA-associated ceRNA networks to reveal potential prognostic biomarkers for glioblastoma multiforme

**DOI:** 10.1186/s12935-020-01488-1

**Published:** 2020-08-15

**Authors:** Xiaolong Zhu, Lan Jiang, Hui Yang, Tianbing Chen, Xingwei Wu, Kun Lv

**Affiliations:** 1 Key Laboratory of Non-coding RNA Transformation Research of Anhui Higher Education Institution, Wannan Medical College, Wuhu, 241001 People’s Republic of China; 2 Non-coding RNA Research Center of Wannan Medical College, Wuhu, 241001 China; 3 Central Laboratory of Yijishan Hospital, Wannan Medical College, Wuhu, 241001 People’s Republic of China

**Keywords:** Competing endogenous RNA, Glioblastoma multiforme, Prognosis

## Abstract

**Background:**

Glioblastoma multiforme (GBM) is the most seriously brain tumor with extremely poor prognosis. Recent research has demonstrated that competitive endogenous RNA (ceRNA) network which long noncoding RNAs (lncRNAs) act as microRNA (miRNA) sponges to regulate mRNA expression were closely related to tumor development. However, the regulatory mechanisms and functional roles of ceRNA network in the pathogenesis of GBM are remaining poorly understood.

**Methods:**

In this study, we systematically analyzed the expression profiles of lncRNA and mRNA (GSE51146 dataset) and miRNA (GSE65626 dataset) from GEO database. Then, we constructed a ceRNA network with the dysregulated genes by bioinformatics methods. The TCGA and GSE4290 dataset were used to confirm the expression and prognostic value of candidate mRNAs.

**Results:**

In total, 3413 differentially expressed lncRNAs and mRNAs, 305 differentially expressed miRNAs were indentified in GBM samples. Then a ceRNA network containing 3 lncRNAs, 5 miRNAs, and 60 mRNAs was constructed. The overall survival analysis of TCGA databases indicated that two mRNAs (C1s and HSD3B7) were remarkly related with the prognosis of GBM.

**Conclusion:**

The ceRNA network may increase our understanding to the pathogenesis of GBM. In general, the candidate mRNAs from the ceRNA network can be predicted as new therapeutic targets and prognostic biomarkers for GBM.

## Background

Glioma is the most frequently occurring primary tumors of the central nervous system (CNS) [1, 2]. According to cell of origin, gliomas are classified astrocytoma, anaplastic astrocytoma, glioblastoma and oligodendrogliomas. Among the gliomas, glioblastoma multiforme (GBM) is the most aggressive and lethal form of cancer and accounts for more than half of brain tumors in adults [3]. The median patient survival time is only 8–15 months after standard treatment with surgery, chemotherapy, radiation and biotherapy [4, 5]. The main reason for the lack of effective treatment and poor prognosis of GBM patients is that the mechanism of GBM is not thoroughly studied [6]. Thus, the precision medicine which based on discovering tumor biomarkers and therapeutic targets are desperately needed to treat GBM. Our research focus on the differentially expressed long non-coding RNAs (lncRNAs), microRNAs (miRNAs), and mRNAs in GBM and the role of competitive endogenous RNAs (ceRNAs) network in the pathogenesis and prognosis of GBM.

Noncoding RNAs (ncRNAs), a class of RNAs with limited protein encoding abilities that are commonly expressed in many tumors including GBM, have received considerable attention [7]. The lncRNAs, a subtype of ncRNA longer than 200 nucleotides, account for 80% of all ncRNAs [8]. The miRNA, a single chain ncRNAs of around 22 nucleotides in length, influence the gene expression by binding to the 3′-UTR of their respective target genes [9]. They are key regulators that mediate tumor development and pathology, such as proliferation, transcription, post-transcriptional modifications, invasion, apoptosis, and cell metabolism [10]. However, the functions of lncRNAs and miRNA in gene expression regulation are not well characterized.

Tumorgenesis is a complex process regulated by various gene networks which one of them is the interaction of lncRNAs with miRNAs, mRNAs or other molecules [11]. Salmena et al. presented the ceRNA network hypothesis which is a novel regulatory mechanism between noncoding RNAs and coding RNAs [12]. The ceRNA is a complicated posttranscriptional regulatory network that lncRNAs, mRNAs, and other RNAs acting as miRNA sponges to competitively bound miRNAs through miRNA response elements (MREs) [13]. These ceRNA modules exert a crucial role in occurrence and development of tumors by regulating the expression levels of various RNAs and proteins [14].

Accumulating data have confirmed that lncRNA–miRNA–mRNA regulatory network exerts a powerful effect in the progression and pathogenesis of many tumors, including GBM [15–17]. Long non-coding RNA taurine upregulated 1 (TUG1) enhances tumor-induced angiogenesis and VEGF expression through inhibiting microRNA-299 in human glioblastoma [18]. LncRNA-SOX2OT-miR-194-5p/miR-122-SOX3-TDGF-1 pathway forms a positive feedback loop and regulates the biological behaviors of glioblastoma stem cells (GSCs) [19]. However, systematic studies for ceRNA networks in GBM are also scarce yet.

In this study, the expression profiles of lncRNAs, miRNAs and mRNAs were systematic analysis between 8 GBM tissues and 8 normal brain tissues from GEO database in total. Then, we performed a ceRNA network associated with GBM, including 3 lncRNAs, five miRNAs, and sixty mRNAs. According to the differential expression and overall survival analysis from the Cancer Genome Atlas (TCGA) databases, two mRNAs (C1s and HSD3B7) were prognostic biomarkers for GBM patients. These candidate genes involved in the ceRNA network may provide clues and ideas for exploring the pathogenesis and accurate diagnostic biomarkers for GBM.

## Materials and methods

### Raw data

The datasets used in the present study were downloaded from the National Center of Biotechnology Information (NCBI) Gene Expression Omnibus (GEO) (https://www.ncbi.nlm.nih.gov/geo/) [20]. The original gene expression profiles were obtained from GSE51146 dataset (no paper published) and GSE65626 dataset. The GSE51146 dataset includes gene expression profiles from 5 glioblastoma multiform biopsy specimens and 5 normal brain tissues. The platform used in GSE51146 dataset is GPL15314 Arraystar Human LncRNA microarray V2.0 (Agilent_033010 Probe Name version). The GSE65626 dataset includes microRNA expression profiles from 3 glioblastoma multiforme specimens (3 tumours and 3 normal tissues). The platform used in GSE65626 dataset is GPL19117 [miRNA-4] Affymetrix Multispecies miRNA-4 Array. The original gene expression profiles of C1s and HSD3B7 were obtained from GSE4290 dataset. The GSE4290 dataset includes gene expression profiles from 81 glioblastoma multiform samples and 23 normal brain tissues [21]. The platform used in GSE4290 dataset is GPL570 Affymetrix Human Genome U133 Plus 2.0 Array.

### Identification of differentially expressed genes

GEO2R (http://www.ncbi.nlm.nih.gov/geo/geo2r/) is an interactive web tool, based on R language limma package [22], that can be used to compare two or more groups of samples to identify differential expression in a GEO series. In the present study, GEO2R was used to filter differentially expressed mRNAs and miRNAs between normal and tumor samples separately in each of the datasets. The false discovery rate (FDR) is a method of conceptualizing the rate of type I errors in null hypothesis testing when conducting multiple comparisons. GEO2R calculates the FDR automatically. The multiple t test was used to detect statistically significant genes at the same time with FDR correction. Fold change (FC) > 2 and P-value < 0.05 were set as the cut-off criteria. Then, probes without a corresponding gene symbol were then filtered.

### Gene function analysis

Gene ontology (GO) enrichment analysis and kyoto encyclopedia of genes and genomes (KEGG) pathway analysis of mRNAs was implemented with DAVID (https://david.ncifcrf.gov/). Briefly, gene identifiers were first converted into their Homo.sapiens Entrez gene IDs using the latest database. If multiple identifiers correspond to the same Entrez gene ID, they will be considered as a single Entrez gene ID in downstream analyses. For each given gene list, pathway and process enrichment analysis was carried out with the following ontology sources: KEGG Pathway, GO biological processes (BP), cellular component (CC) and molecular function (MF). The species was limited to Homo.sapiens and all genes in the genome were used as the enrichment background. More specifically, p-values are calculated based on accumulative hyper geometric distribution, q-values are calculated using the Banjamini-Hochberg procedure to account for multiple testing [23]. Kappa scores were used as the similarity metric when performing hierarchical clustering on the enriched terms and then sub-trees with similarity > 0.3 are considered a cluster. The most statistically significant term within a cluster is chosen as the one representing the cluster.

### WGCNA analysis

Samples clustering were performed to demonstrate the relationship between expression profile and clinical traits. After raw data preprocessing, weighted gene co-repression network analysis (WGCNA) were performed to identify significant gene modules according to a previously described algorithm [24]. Probe sets were first filtered based on the variance of expression value across all samples. Probe sets with duplicated gene symbols were also removed based on expression variance. The R package WGCNA [25] was applied for this analysis. Briefly, Person’s correlation coefficients were calculated for selected genes in a pair wise manner yielding a similarity matrix (Sij). The soft threshold (power) was set as 12. The matrix was transformed into an adjacency matrix (aij) using a power function using formula aij = Power (Sij, β) ≡ |Sij| β. Average linkage hierarchical clustering was then performed to identify modules of densely interconnected genes. Network interconnectedness was measured by calculating the topological overlap using the TOM dist function with a signed TOM-Type. Average hierarchical clustering was performed to group the genes based on the topological overlap dissimilarity measure (1-TOM) of their connection strengths. Network modules were identified using a dynamic tree cut algorithm with minimum cluster size of 30 and merging threshold function at 0.25. Genes that were not assigned to specific modules were assigned to the color grey.

### Prediction of the mRNA-miRNA-lncRNA interactions

The interactions between the differentially expressed miRNAs and differentially expressed mRNAs were predicted using miRWalk 3.0 (http://mirwalk.umm.uni-heidelberg.de/), which integrated the prediction results of both TargetScan [26] and miRDB [27], and the score ≥ 0.95 was considered as cutoff criterion for the prediction analysis in miRWalk. Only the interacion of miRNA and mRNA with opposite expression was included in the present study. The interaction between miRNA and lncRNA was predicted by using DIANA-LncBase v2.0 [28] and the score ≥ 0.4 was considered as cut off criterion for the prediction analysis in the prediction module of LncBase.The predicted targets were intersected with DEGs, the miRNAs, and mRNAs were selected for construcion of miRNA-mRNA regulatory network. Cytoscape software (version 3.40) was used to visualize the regulatory network.

### Patients and tissue samples

The use of these archival tissues in this study was reviewed and approved by the Ethics Committee of the Yijishan Hospital of Wannan Medical College. Archival human non-glioma patient samples and glioma tissue samples were obtained from Department of Neurosurgery, Yijishan Hospital of Wannan Medical College. The samples taken during the surgery were immediately frozen in − 80 °C and then used for RNA isolation. Gliomas were graded according to the WHO classification of tumors [29].

### Cell lines and culture

The human normal glial cell line (HEB) and human GBM cell lines (U87MG, U251) which used in this study were obtained from American Type Culture Collection (Manassas, VA). All cell lines were cultured in Dulbecco’s modified eagle medium (DMEM) (Hyclone, GE Healthcare Life Sciences, Logan, UT, USA) at 37 °C in a humidified atmosphere containing 5% CO_2_. All media were supplemented with 10%(v/v) fetal bovine serum (FBS) (Gibco, Life Technologies, Grand Island, NY). Briefly, When the human cells are near the end of exponential growth (roughly 70% to 90% confluent), remove and discard culture medium and wash the cells with PBS. Add Trypsin–EDTA solution to flask and until cell layer is dispersed. Add DMEM medium and aspirate cells to EP tube. Resuspend the cell pellet and add appropriate aliquots of the cell suspension to new flask. Incubate cultures at 37 °C.

### RNA isolation and real-time qPCR analysis

Total RNA was isolated from the frozen brain tissues and cells using TRIzol reagent (Ambion, life technologies, Carlsbad, CA, USA) according to the previous description [30]. For mRNA detection, total RNA was used to reverse transcribe C1s and HSD3B7 by a Revert Aid First Strand cDNA Synthesis Kit (Thermo Scientific, Vilnius, Lithuania, USA). RT-qPCR was performed using Quanti Nova™ SYBR^®^ Green PCR Kit (Qiagen, Hilden, Germany). Primer sequences for qPCR: C1s Forward, AGGCACCTCTTCCGACTACAACC; C1s Reverse, CCTTGAGGCGA ACAGCACGATC; HSD3B7 Forward, CTACTGGCTGCTGGTGTTCCTG; HSD3B7 Reverse, CTGACGGTGAAGGTGGTGTTGG. The amplification was performed on a Bio-Rad CXF96 PCR detection system (Bio-Rad, Hercules, CA, USA). The expressions of the mRNAs were normalized to GAPDH which used as endogenous controls. The relative gene expression levels were calculated with the comparative cycle threshold (2^−ΔΔCt^) method. For RT-PCR analysis of tissues, results were analyzed also using the 2^−ΔΔCt^ method. Fold change of RT-PCR was presented as 2^−ΔΔCt^, where ΔCt = CtRNAs − CtGAPDH. Then select the ΔCt value of a tissue as a control. ΔΔCt _tissues_ = ΔCt _control_ − ΔCt _tissues_ and Fold change (Relative mRNA expression) = 2^−ΔΔCt^.

### Statistical analyses

All procedures were performed in triplicate. The Data were presented as the mean ± standard deviation (SD). Statistical analyses were performed using GraphPad Prism 5 (La Jolla, California, USA). The significance of the differences was determined by Student’s t-test. Differences were considered statistically significant at *p < 0.05, **p < 0.01 and ***p < 0.001.

Supplementary methods are described in Additional file 1.

## Results

### Screening differentially expressed lncRNAs, miRNAs and mRNAs

**Fig. 1:**
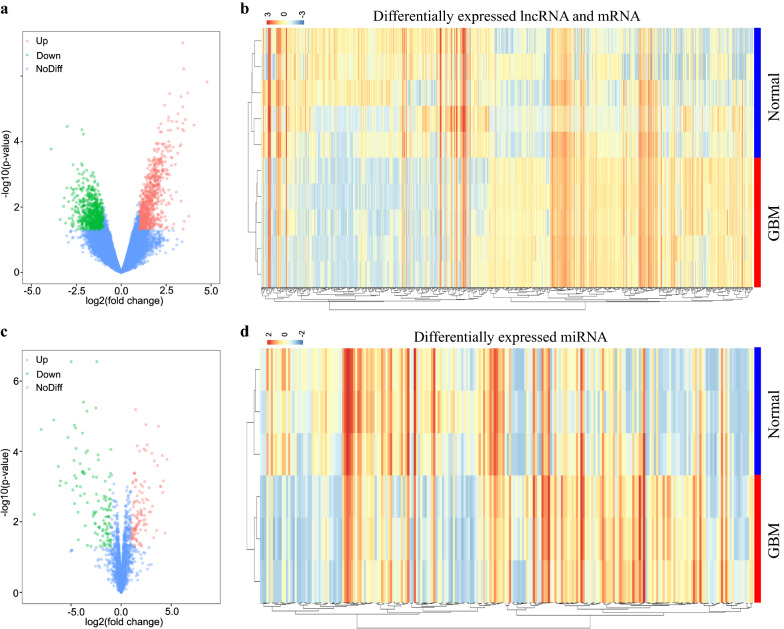
Analysis of differentially expressed lncRNAs, miRNAs and mRNAs in GBM from GEO databases. **a** Volcano plot depicting differentially expressed lncRNAs and mRNAs between 5 paired GBM and normal samples. **b** The heatmap plot of differentially expressed lncRNAs and mRNAs from **a**. **c** Volcano plot depicting differentially expressed miRNAs between 3 paired GBM and normal samples. Volcano plot depicting. **d** The heatmap plot of differentially expressed miRNAs from **c**. The normal represents normal brain tissues. *Up* upregulation, *Down* downregulation, *NoDiff* no difference

Firstly, the genome wide mRNA and lncRNA expression profiles in GBM were analyzed in GSE51146 dataset (including 5 GBM biopsy specimens and 5 normal brain tissues) by GEO2R. Fold change (FC) > 2 and P-value < 0.05 were set as the cut-off criteria. Compared with normal brain, we found that 1531 and 1882 genes were identified significantly up- and down-regulated in GBM, respectively (Fig. 1a, b) (Additional file 2: Table S1). Using the same method with miRNAs, there were 305 (including 152 up and 153 down) differentially expressed miRNAs between GBM and normal brain groups from GSE65626 dataset (including 3 GBM specimens and 3 normal brain tissues) (Fig. 1c, d) (Additional file 3: Table S2).

### Co-expression analysis of lncRNA and mRNA

**Fig. 2:**
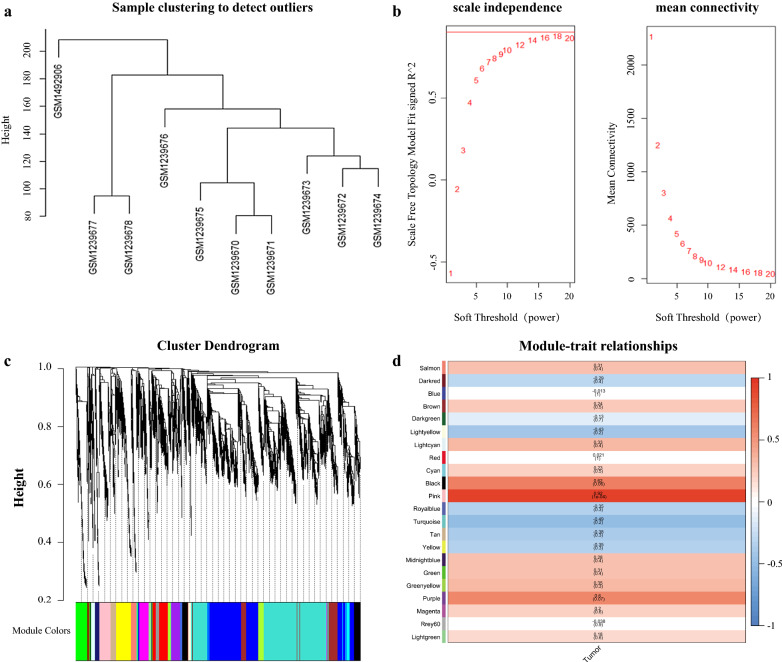
Construction of co-expression network of lncRNA and mRNA by WGCNA. **a** Sample clustering to detect outliers based on lncRNA and mRNA data. All the samples were in the clusters. **b** Analysis of network topology for various soft-threshold powers. The soft threshold (power) was set as 12. **c** Cluster dendrogram of all aberrantly expressed lncRNAs and mRNAs was constructed based on the topological overlap dissimilarity measure (1-TOM) of their connection strengths. There are 22 co-expression modules which shown in different colors were constructed. **d** Analysis of module-trait relationships. Each row corresponds to a module eigengene, column to a trait. In each cell, upper number is the correlation coefficient (R) and lower number is the corresponding P-value

To explore biologic function of lncRNAs and mRNAs in GBM, we need to define the regulatory relationship between lncRNAs and mRNAs. Because lncRNAs could compete with miRNA target mRNAs, we just focused on an lncRNA–mRNA competing interaction pair which the expression of lncRNA and mRNA is positively correlated. We analyzed the co-expression of lncRNA and mRNA from GSE51146 dataset by WGCNA package. As shown in Fig. 2, a heatmap with correlation coefficient (R) and significant difference (P-value) showed the correlation between module eigengenes with GBM. Finally, we get the co-expression Pink module (R = 0.92, p = 0.0001) which the lncRNAs and mRNAs in the module were significantly correlation with GBM.

**Fig. 3:**
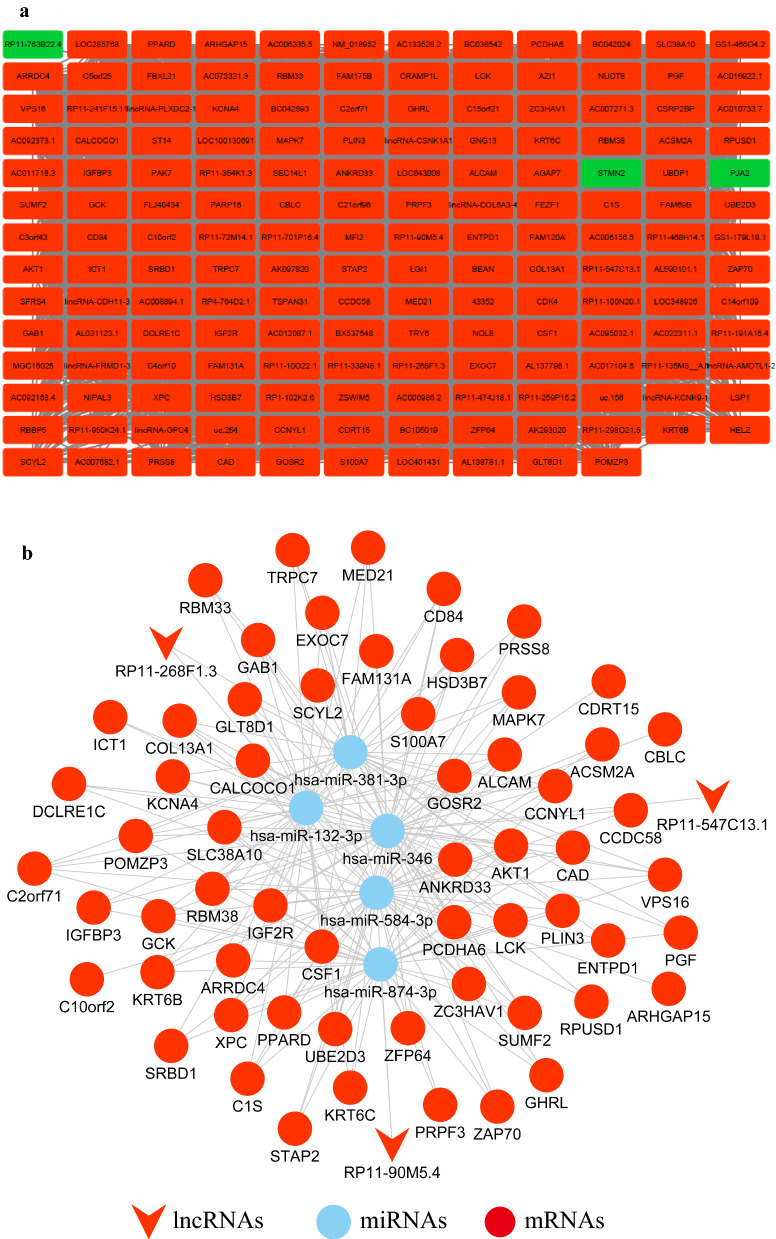
Co-expression of Pink module and lncRNA-miRNA-mRNA network. **a** Construction of co-expression network of Pink modules between lncRNAs and mRNAs. **b** The lncRNA-miRNA-mRNA ceRNA network based on the co-expression Pink module and differentially expressed miRNAs in GEO databases. Red arrowheads, lncRNAs; Blue circles, miRNAs; Red circles, mRNAs

Then we tried to build the co-expression network diagram for the Pink module. The screening threshold for co-expression relationships is set to weight > 0.02; meanwhile, lncRNAs and mRNAs were differentially expressed genes and had the same expression trend. As shown in Fig. 3a, the co-expression network of the pink module was constructed. In total, 5928 potential lncRNA and mRNA pairs were constructed (Additional file 4: Table S3). Then we computed the all genes’ node degree which is a basic topological feature of co-expression network (Additional file 5: Table S4). The node degree can directly reflect the importance of the gene in the network. A gene with a relatively large degree may have more important clinical and biological value [31]. As a result, 7 lncRNAs were obtained, including RP11-268F1.3, RP11-547C13.1, lincRNA-GPC4, RP11-90M5.4, RP11-339N8.1, RP4-764D2.1, GS1-466O4.2, which degrees were over 100.

### The lncRNAs, miRNAs and mRNAs regulatory networks in GBM

To further study the influence of 7 lncRNAs regulation on mRNAs expression in GBM, we identified lncRNA-miRNA-mRNA ceRNA modules by integrating matched expression profiles of lncRNAs, miRNAs and mRNAs (Additional file 6: Fig. S1). The miRNA interacted with lncRNA were identified by intersecting the miRNA which predicted by using DIANA-LncBase v2.0 and differentially expressed miRNAs from Fig. 1. Then we used miRWalk 3.0 to identify the mRNAs which targeted with miRNAs acquired from the above analysis. Three lncRNA-miRNA-mRNA ceRNA modules were performed after the target mRNAs of miRNA intersect with the mRNAs in the co-expression network of the Pink module from Fig. 3 (Fig. 3b) (Additional file 7: Table S5). In the first module, lncRNA RP11-547C13.1 competed with 51 mRNAs to target with miR-346. In the second module, lncRNA RP11-268F1.3 and 47 mRNAs competed with 2 miRNAs (miR-132-3p and miR-381-3p). While the third module contained single lncRNA (RP11-90M5.4), 2 miRNAs (miR-874-3p and miR-584-3p) and 52 mRNAs.

### Gene ontology and KEGG pathway analyses

**Fig. 4:**
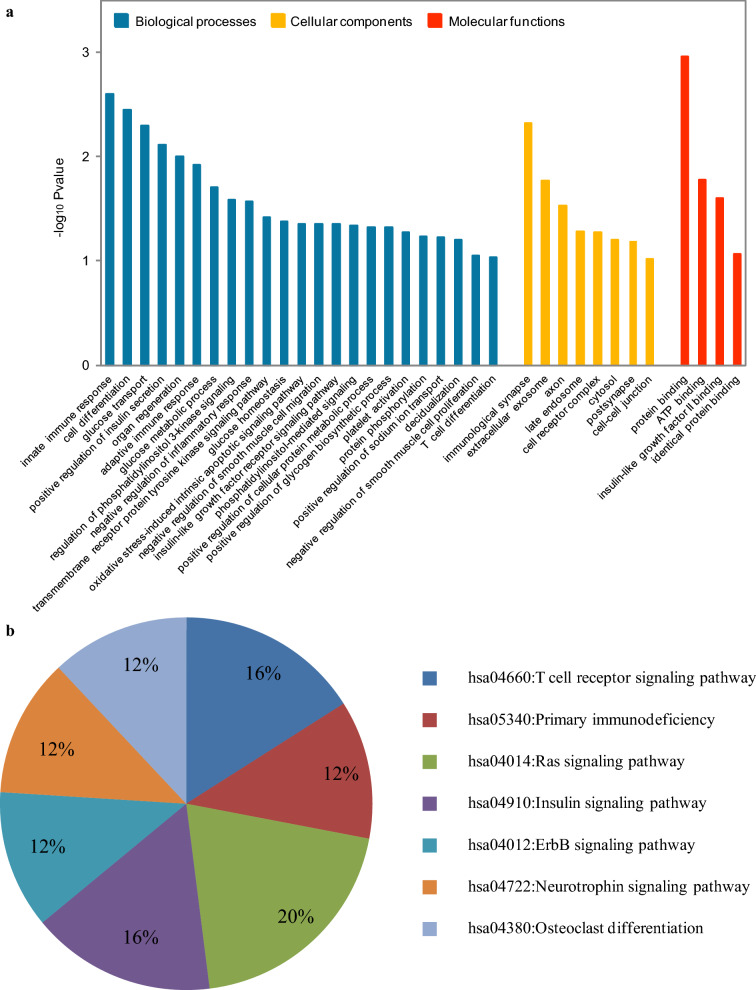
Pathway enrichment analysis of mRNAs involved in the ceRNA network. **a** GO pathway analysis for the mRNAs involved in the ceRNA network. The horizontal axis is the GO pathway term; the vertical axis represents significant difference of GO pathway enrichment. **b** KEGG pathway analysis for the mRNAs involved in the ceRNA network. The proportion in the pie chart is the percentage of genes in the KEGG pathway

We further investigated the biological function and pathways of mRNAs in ceRNA network by GO and KEGG pathway analysis (Additional file 8: Table S6). As shown in Fig. 4a, mRNAs in ceRNA network were classified as 23 biological processes (BP) terms, 8 cellular components (CC) terms and 4 molecular functions (MF) terms using GO enrichment analysis. The significantly enriched BP terms were related to innate immune response, cell differentiation, glucose transport, positive regulation of insulin secretion. The most significantly enriched CC term was immunological synapse, and the most significantly enriched term under the MF classification was protein binding. KEGG pathway analysis revealed that the mRNAs in ceRNA network were mainly involved in the T cell receptor signaling pathway, primary immunodeficiency and Ras signaling pathway (Fig. 4b).

### Clinical validation of candidate mRNA

**Fig. 5:**
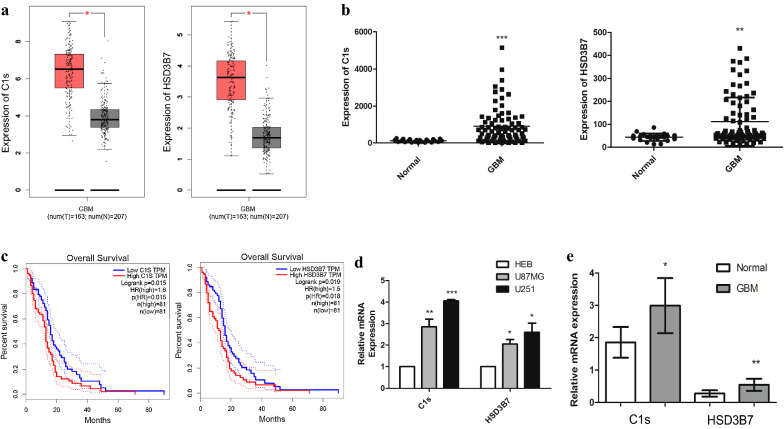
The expression and overall survival of C1s and HSD3B7 in ceRNAs network. **a** The differences between expression levels of C1s and HSD3B7 in GBM and normal samples from TCGA database. **b** The differences between expression levels of C1s and HSD3B7 in GBM and normal samples from GEO database. **c** The relationships between the expression levels of C1s or HSD3B7 and overall survival of patients with GBM from TCGA database. The horizontal axis is the survival time (months); the vertical axis represents survival rate. **d** The differences between expression levels of C1s and HSD3B7 in GBM and normal glial cells. RT-qPCR detected the mRNA expression of C1s and HSD3B7 in HEB, U87MG, U251 cells. **e** The differences between expression levels of C1s and HSD3B7 in GBM and normal samples from Yijishan Hospital. RT-qPCR detected the mRNA expression of C1s and HSD3B7. Data are mean ± SD from three independent experiments.*p < 0.05, **p < 0.01, ***p < 0.001

To test whether the mRNAs in ceRNA network had clinical significance,we verified the gene expression and its relationship with patient overall survival by using the GBM samples from TCGA and GEO database. We found that the expression of C1s and HSD3B7 were significantly increased in GBM vs. controls and correlated with overall survival in patients simultaneously (Fig. 5a–c). By TCGA database, the expression of C1s or HSD3B7 in GBM based on patient’s age and gender were presented in (Additional file 9: Fig. S2). RT-qPCR showed that the expression levels of C1s and HSD3B7 in GBM cell lines were significantly higher than that in normal glial cell line (Fig. 5d). Then we further determined the expression of C1s and HSD3B7 in GBM and normal brain tissues which collected from the Yijishan Hospital of Wannan Medical College. The results shown that the expression of C1s and HSD3B7 were up-regulated in GBM tissues as compared to the normal brain tissues (Fig. 5e) (Additional file 10: Fig. S3). These results suggest that high expression of C1s and HSD3B7 may play biomarkers for GBM diagnosis.

We then investigated the lnRNA and miRNA involved in the regulation of C1s and HSD3B7. According to the ceRNA network, miR-132-3p, miR-346, miR-584-3p and miR-874-3p may target C1s, and miR-132-3p, miR-346, miR-381-3p and miR-584-3p may target HSD3B7. Therefore, we detected the expression levels of C1s and HSD3B7 in GBM cells transfected with miRNA mimics and NC. Compared with the NC, overexpression of miR-132-3p, miR-346, miR-584-3p and miR-874-3p significantly increased the expression level of C1s. However, the expression of HSD3B7 was almost unaffected by the interacting miRNA (Additional file 11: Fig. S4 A and B). To confirm the connection between the lnRNA and miRNA which regulated C1s, we constructed luciferase reporters containing full-length lncRNA RP11-268F1.3, RP11-547C13.1, RP11-90M5.4. The miR-132-3p, miR-346 and miR-874-3p mimics significantly reduced the luciferase activities of the corresponding lncRNA reporters (Additional file 11: Fig. S4 C and D). Expression analysis indicated that C1s was decreased upon RP11-268F1.3, RP11-547C13.1 and RP11-90M5.4 knockdown (Additional file 11: Fig. S4 E). Collectively, these data indicated that lncRNA RP11-268F1.3, RP11-547C13.1 and RP11-90M5.4 serves as ceRNA through direct binding with corresponding miRNAs in GBM.

## Discussion

GBM is the most malignant type of CNS tumors with complex biology and poor prognosis. The study on the molecular mechanism of GBM is of great significance to the treatment and survival of patients. With the application of high-throughput technology and the progress of bioinformatics technology, the underlying mechanism and efficient biomarkers of GBM have been greatly improved [32]. In this study, the transcriptional expression and clinically relevant pathological features of lnRNAs, miRNAs and mRNAs were collected from the TCGA and GEO databases. Based on comprehensive integration of the lncRNA, miRNA and mRNA data, ceRNA networks were constructed and two candidate biomarkers (C1s and HSD3B7) that may be associated with GBM prognosis were identified.

One of the mRNAs that we screened from the prognostic ceRNA network was C1s which is a major constituent of the human complement subcomponent C1. C1s interacts with two other complement components C1r and C1q to form the C1 complex (C1qC1r_2_C1s_2_). C1 complex, the first component of the serum complement system, responds to clear pathogens and initiate inflammation by leading to the production of opsonins and anaphylatoxins [33, 34]. Previous study shows that C1s is expressed in the liver, brain and kidney [35]. It has been demonstrated that C1s deficiency was associated with ICR-Derived Glomerulonephritis (ICGN) and systemic lupus erythematosus [36, 37]. However, little research has been done on the function of C1s in tumors and gliomas. In this study, we have proved that the expression of C1s was upregulated in GBM tissues and that its overexpression level presented a poor prognosis in patients with GBM (Fig. 5).

Among the prognostic differentially expressed mRNAs, HSD3B7 encodes an enzyme which is involved in the initial stages of the synthesis of bile acids from cholesterol and a member of the short-chain dehydrogenase/reductase superfamily [38]. Mutations in this gene are associated with a congenital bile acid synthesis defect which leads to neonatal cholestasis, but reduces the risk of late-onset Parkinson’s disease [39, 40]. 7α25HC, an intermediate product of bile acid synthesis, is degraded by HSD3B7. In astrocytes, EBI2 is activated by 7α25HC and regulates ERK phosphorylation, Ca^2+^ signaling, as well as astrocyte cell migration [41, 42]. Studies have shown that EBI2 might be involved in the recruitment of monocytes and macrophages towards GBM [43]. Combined with clinical data, we suspect that HSD3B7 may play a role in GBM by regulating EBI2 activity.

In addition to mRNAs, we screened 3 lncRNAs (lncRNA RP11-268F1.3, RP11-547C13.1, RP11-90M5.4) and five miRNAs (miR-132-3p, miR-346, miR-381-3p, miR-584-3p and miR-874-3p) through the ceRNA network. In our model, lncRNA RP11-268F1.3, RP11-547C13.1 and RP11-90M5.4 functions as a posttranscriptional modulator by directly interacting with miR-132-3p, miR-346 and miR-874-3p, respectively. Thereby finally lncRNA regulates the miRNA target C1s. As all we know, miRNAs are important post-transcriptional regulators of mRNAs and negatively regulate gene expression by either inhibiting protein translation or degrading mRNA [9]. Current research reveals that these three miRNAs (miR-132-3p, miR-346 and miR-584-3p) could affect multiple functions of glioma [44–46]. The other two miRNAs (miR-381-3p and miR-874-3p) also have been found to involve in biological processes of most tumors except gliomas [47, 48].

In this study, GO and KEGG were employed to analyze the biological function and pathways of mRNAs in ceRNA network. We found that most pathways were related with cell immunity. In addition, the mRNAs also significantly enriched in tumor related pathways, such as cell differentiation and Ras signaling pathway (Fig. 4 and Additional file 8: Table S6). The biological meaning of cell differentiation refers to the process by which cells of the same origin gradually produce cell groups with different morphological structures and functional characteristics. Highly malignant tumor cells are generally associated with abnormal differentiation and immaturity [49]. RAS encodes a family of small GTPases that includes; NRAS, HRAS and KRAS. The GTP-bound RAS, as an active form of RAS, is important regulator of tumorgenesis by activating the PI3K/AKT/MTOR or RAF/MEK/ERK pathways [50].

We identified many GBM related lncRNAs, miRNAs, and mRNAs from the ceRNA network, but the relationship between the ceRNA network and GBM remain not clear. Therefore, more experiments and clinical practice were still needed to verify the effect of ceRNA network in GBM. We insist that the genes in the ceRNA network are critical for GBM diagnosis and treatment because the network was a result of integrated microarray and experimental evidence. In the future, the ceRNA network will be improved with the better databases, optimization of algorithms, and increased samples. Next, we will explore the functions of ceRNAs in GBM.

## Conclusions

In summary, a ceRNA network was successfully established after screening differentially expressed lncRNAs, miRNAs and mRNAs in GBM from the GEO database. Overall survival analysis of TCGA database indicated that two mRNAs (C1s and HSD3B7) from the ceRNA network were significantly correlated with the prognosis of GBM patients. Furthermore, the GEO database and brain tissues of our hospital confirmed that the expression levels of C1s and HSD3B7 in GBM tissues and cells were higher than those in normal brain tissues and cells. Therefore, C1s and HSD3B7 can be further used as potential prognostic biomarker for GBM and promising targets for treatment.

## Supplementary information

**Additional file 1.** Additional methods.

**Additional file 2: Table S1.** Differentially expressed lncRNAs and mRNAs between GBM samples and normal brain samples in GSE51146.

**Additional file 3: Table S2.** Differentially expressed miRNAs between GBM samples and normal brain samples in GSE65626.

**Additional file 4: Table S3.** Co-expression of Pink module between lncRNAs and mRNAs.

**Additional file 5: Table S4.** The node degree of lncRNAs and mRNAs in Pink module.

**Additional file 6: Figure S1**. Workflow plot of construction of ceRNA network.

**Additional file 7: Table S5.** The lncRNA-miRNA-mRNA ceRNA network.

**Additional file 8: Table S6.** GO and KEGG enrichment for the mRNAs involved in the ceRNA network.

**Additional file 9: Figure S2**. Expression of C1s and HSD3B7 in GBM based on patients. (A) Expression of C1s in GBM based on patient’s age. (B) Expression of C1s in GBM based on patient’s gender. (C) Expression of HSD3B7 in GBM based on patient’s age. (D) Expression of HSD3B7 in GBM based on patient’s gender.

**Additional file 10: Figure S3**. C1s and HSD3B7 are up-regulated in GBM tissues. Western blot detection of C1s and HSD3B7 expression in 12 cases of GBM tissue (T) and normal tissue (N).

**Additional file 11: Figure S4**. lncRNA-miRNA-mRNA ceRNA network verification. (A) Expression levels of C1s were detected by RT-PCR in miR-132-3p, miR-346, miR-584-3p, miR-874-3p-overexpressed U251 cells. (B) Expression levels of HSD3B7 were detected by RT-PCR in miR-132-3p, miR-346, miR-381-3p, miR-584-3p-overexpressed U251 cells. (C) Putative binding sequence between lncRNAs and miRNAs. (D) Relative luciferase activities of reporters containing lncRNA RP11-268F1.3, RP11-547C13.1, RP11-90M5.4. (E) RT-PCR analysis showed that C1s was downregulated in lncRNA RP11-268F1.3, RP11-547C13.1, RP11-90M5.4-silenced U251 cells. Data are mean ± SD from three independent experiments.* p < 0.05, ** p < 0.01, *** p < 0.001.

## Data Availability

The data used to support the findings of this study are included within the article.

## Abbreviations

GBM: Glioblastoma multiforme
TCGA: The Cancer Genome Atlas
GEO: Gene expression omnibus
ceRNA: Competing endogenous RNA
lncRNA: Long noncoding RNA
miRNA: microRNA
WGCNA: Weighted gene co-repression network analysis
GO: Gene ontology
KEGG: Kyoto encyclopedia of genes and genomes
BP: Biological processes
CC: Cellular components
MF: Molecular functions
FC: Fold change
MRE: miRNA response element
FDR: False discovery rate
CNS: Central nervous system
ncRNAs: Noncoding RNAs
GSCs: Glioblastoma stem cells
DEGs: Differentially expressed genes
SD: Standard deviation
