# Supplementary material for: Analyzing the lncRNA, miRNA, and mRNA-associated ceRNA networks to reveal potential prognostic biomarkers for glioblastoma multiforme
Source: Cancer Cell Int. 2020 Aug 15;20:393. doi: 10.1186/s12935-020-01488-1 (PMC7429694; doi:10.1186/s12935-020-01488-1)
Supplement: Supplementary file 1 — Additional file 1. Additional methods. [file 12935_2020_1488_MOESM1_ESM.docx]

**Additional file 1: Additional Methods**

**lncRNA interference and miRNA overexpression**

The lncRNA smart silencer and miRNA mimics were purchased from RiboBio (Guangzhou, China) and transfected into cells by using riboFECT™ CP Transfection Kit (RiboBio) at a final concentration of 100 nM according to the manufacturer's protocol.

**Dual-luciferase assay**

Luciferase reporters were generated by cloning lncRNA RP11-268F1.3, RP11-547C13.1, RP11-90M5.4 into psiCHECK2 vector. Then, U251 cells were cotransfected with reporters and miRNA mimics (RiboBio) using the Lipofectamine® 3000 (Invitrogen, USA). Cell lysates were harvested 48 hr after transfection, and Luciferase activity levels were measured using the Dual-Luciferase Reporter Assay System (Promega, USA) with GloMax-Multi+ Microplate Multimode Reader (Promega) according to the manufacturer’s instructions.

**Western blots**

Expression of C1S (Rabbit Polyclonal; Proteintech, Rosemont, USA) and HSD3B7 (Rabbit Polyclonal; Invitrogen, USA) was determined by western blots. The proteins were resolved by 10% SDS–PAGE gel and transferred to a nitrocellulose membrane which incubated with primary and secondary antibody. Protein bands were captured by Tanon 5200 Chemiluminescence Imaging System (Shanghai, China).
