# Supplementary figures and images for: Analyzing the lncRNA, miRNA, and mRNA-associated ceRNA networks to reveal potential prognostic biomarkers for glioblastoma multiforme
Source: Cancer Cell Int. 2020 Aug 15;20:393. doi: 10.1186/s12935-020-01488-1 (PMC7429694; doi:10.1186/s12935-020-01488-1)

Figure S1

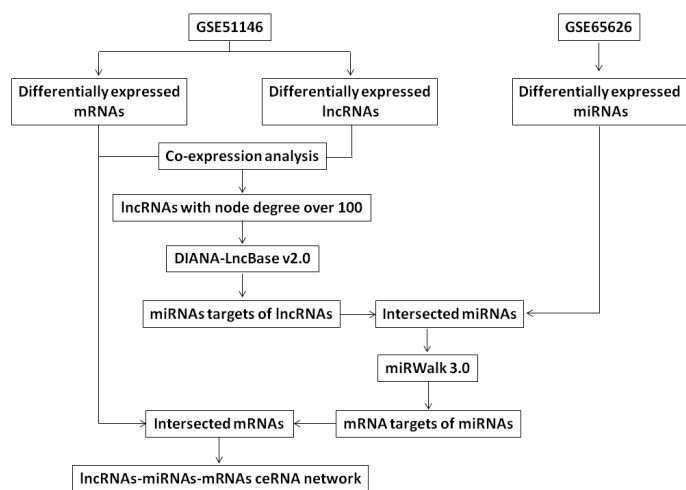

Supplement: Supplementary file 6 — Additional file 6: Figure S1. Workflow plot of construction of ceRNA network. [file 12935_2020_1488_MOESM6_ESM.pdf]

Figure 1

A.

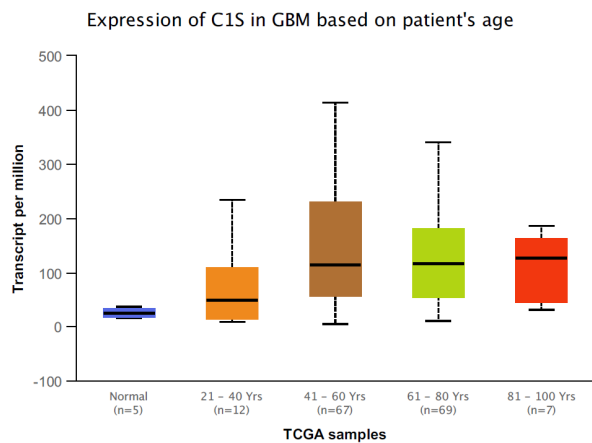

B.

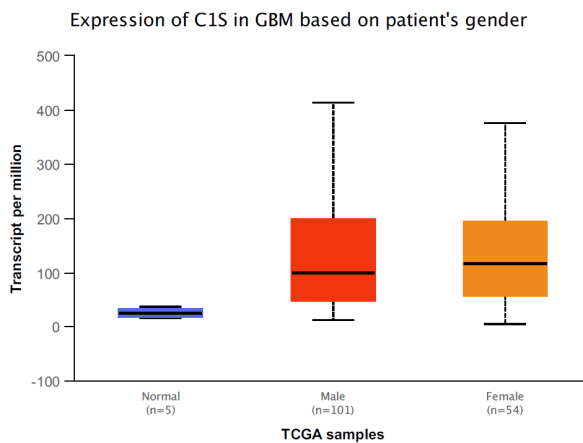

C.

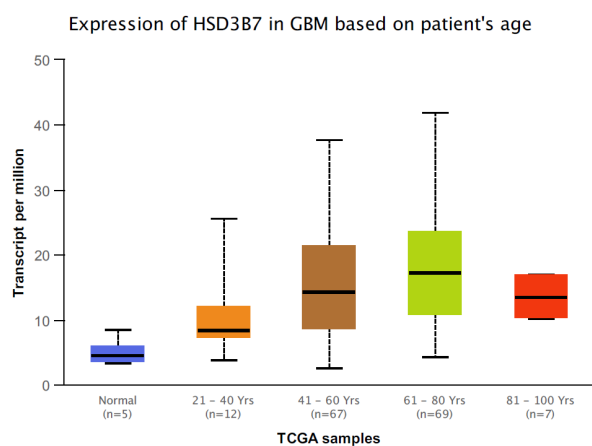

D.

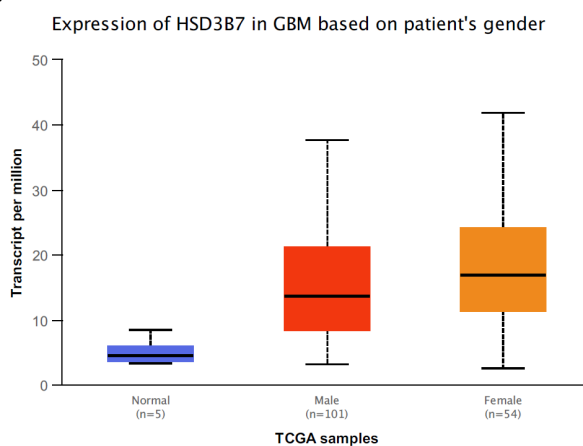

Supplement: Supplementary file 9 — Additional file 9: Figure S2. Expression of C1s and HSD3B7 in GBM based on patients. (A) Expression of C1s in GBM based on patient’s age. (B) Expression of C1s in GBM based on patient’s gender. (C) Expression of HSD3B7 in GBM based on patient’s age. (D) Expression of HSD3B7 in GBM based on patient’s gender. [file 12935_2020_1488_MOESM9_ESM.pdf]

Figure S3

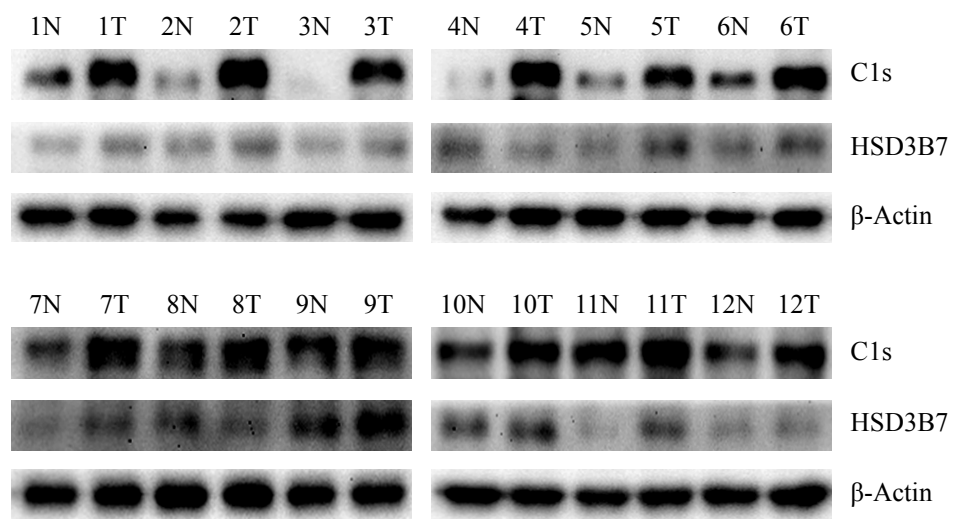

Supplement: Supplementary file 10 — Additional file 10: Figure S3. C1s and HSD3B7 are up-regulated in GBM tissues. Western blot detection of C1s and HSD3B7 expression in 12 cases of GBM tissue (T) and normal tissue (N). [file 12935_2020_1488_MOESM10_ESM.pdf]

Figure S4

A.

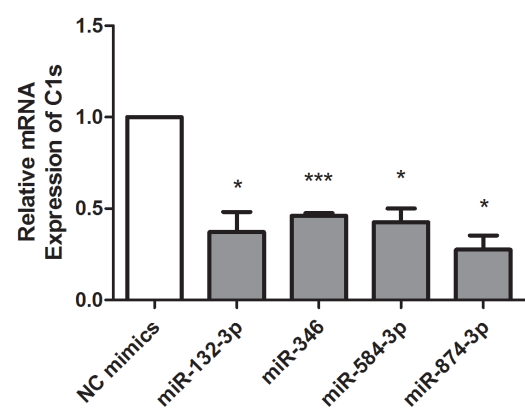

B.

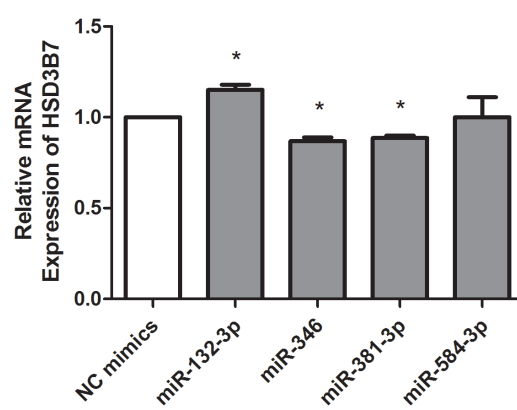

C.

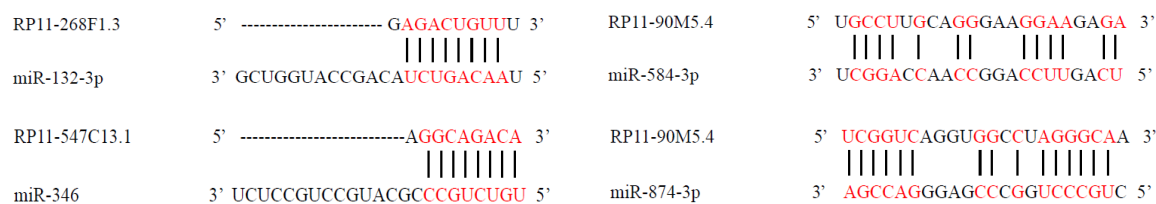

D.

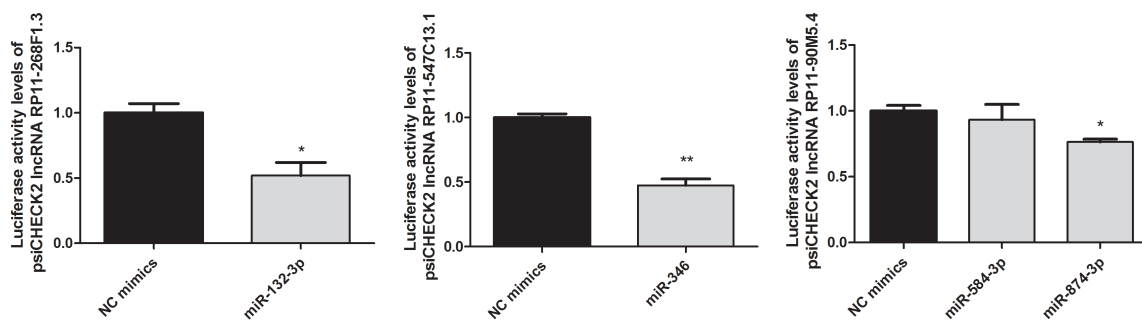

E.

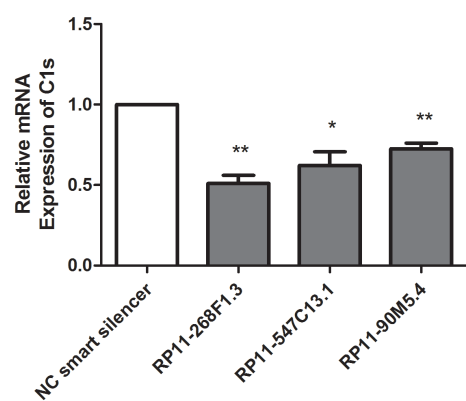

Supplement: Supplementary file 11 — Additional file 11: Figure S4. lncRNA-miRNA-mRNA ceRNA network verification. (A) Expression levels of C1s were detected by RT-PCR in miR-132-3p, miR-346, miR-584-3p, miR-874-3p-overexpressed U251 cells. (B) Expression levels of HSD3B7 were detected by RT-PCR in miR-132-3p, miR-346, miR-381-3p, miR-584-3p-overexpressed U251 cells. (C) Putative binding sequence between lncRNAs and miRNAs. (D) Relative luciferase activities of reporters containing lncRNA RP11-268F1.3, RP11-547C13.1, RP11-90M5.4. (E) RT-PCR analysis showed that C1s was downregulated in lncRNA RP11-268F1.3, RP11-547C13.1, RP11-90M5.4-silenced U251 cells. Data are mean ± SD from three independent experiments.* p < 0.05, ** p < 0.01, *** p < 0.001. [file 12935_2020_1488_MOESM11_ESM.pdf]
